# Chairside 3-D printed impression trays: a new approach to increase the accuracy of conventional implant impression taking? An in vitro study

**DOI:** 10.1186/s40729-023-00516-9

**Published:** 2023-12-06

**Authors:** Alexander Schmidt, Cara Berschin, Bernd Wöstmann, Maximiliane Amelie Schlenz

**Affiliations:** Department of Prosthodontics, Dental Clinic, Justus Liebig University, Schlangenzahl 14, 35392 Giessen, Germany

**Keywords:** 3-D printing, CAD–CAM, Dental implants, Dental impression technique, Manufactured materials, Open impression technique, Impression accuracy, Precision, Trueness, Full-arch implant case

## Abstract

**Purpose:**

A high transfer accuracy of the intraoral implant position to a model is required, to manufacture implant-supported restorations. However, clinically relevant deviations persist between the intraoral implant position and the model obtained, even for the benchmark conventional custom implant impressions with polyether. Thus, new approaches using 3-D printed impression trays may increase the transfer accuracy of implant impressions. The ability to adjust parameters such as the thickness of the layers and the influence of the openings in the impression tray could potentially affect accuracy.

**Methods:**

Four different types of impression trays (*n* = 10 for each group) for the conventional impression technique were investigated: conventional custom impression tray, customized foil tray, chairside 3-D printed impression tray with the SHERA system, and the Primeprint system using an implant master model with four implants in the posterior region and a reference cube. After plaster model casting, all models were measured using a coordinate measuring machine, and the deviation from the reference dataset was determined. A statistical ANOVA analysis was performed (*p* < 0.05).

**Results:**

Chairside 3-D printed impression trays showed the best results, followed by conventional custom impression trays. Implant impressions obtained using a customized foil tray exhibited the lowest accuracy. Statistically significant differences were observed between 3-D printed impression trays and conventional custom impression and customized foil trays (*p* < 0.05). Whereas, the implant position did not have any significant influence on accuracy (*p* > 0.05).

**Conclusions:**

Chairside 3-D printed impression trays significantly increase the transfer accuracy for implant impression taking.

**Graphical Abstract:**

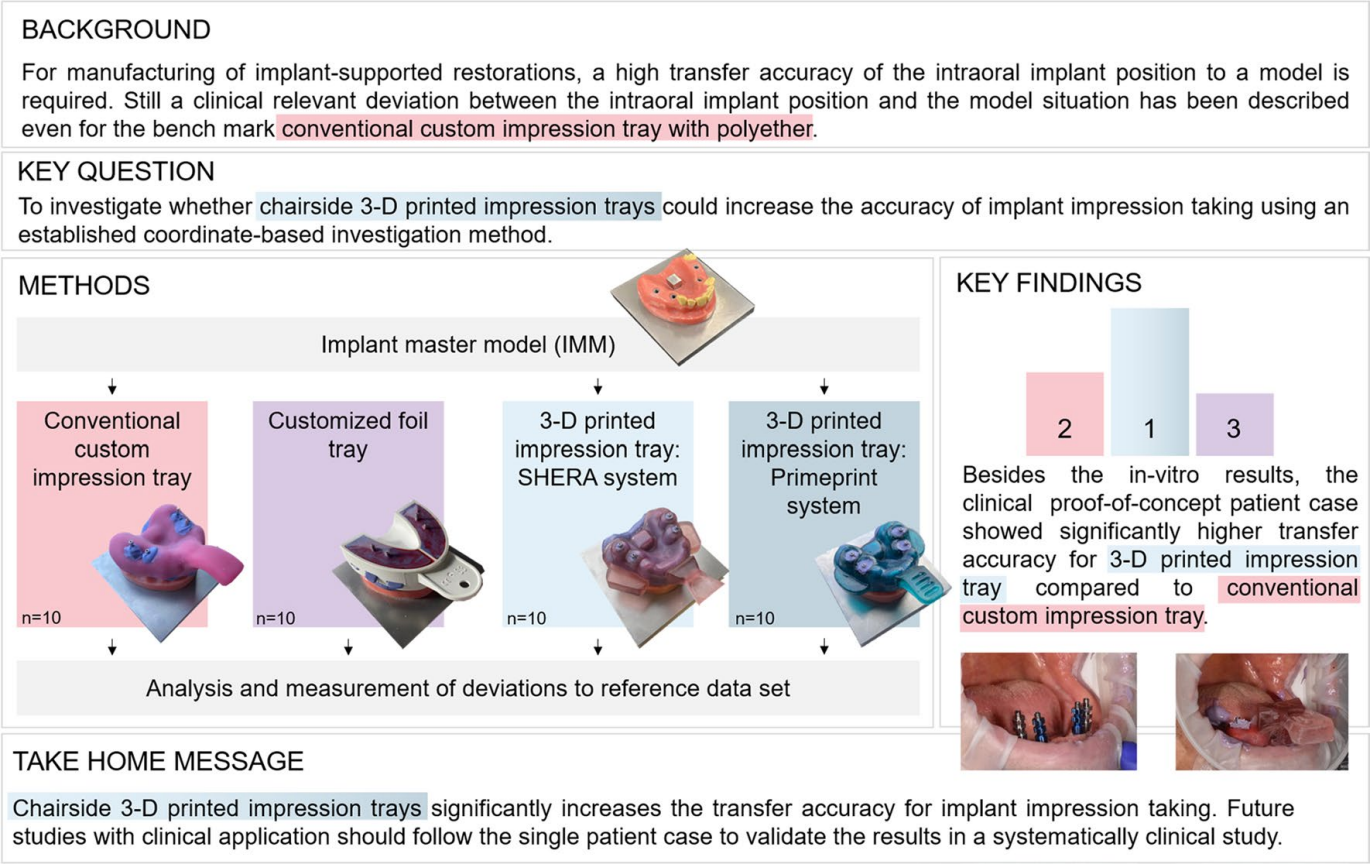

## Background

To manufacture implant-supported restorations, the transfer of the intraoral implant position to a virtual or plaster model is essential [1, 2]. Even though recent data exhibited a huge improvement regarding the accuracy of implant impression taking, especially in the digital workflow using scanbodies and intraoral scanners, a clinically relevant deviation persists between the intraoral implant position and the model obtained, even for the benchmark conventional custom implant impressions with polyether [3–5]. In contrast to natural teeth, implants have an inherent mobility of only 8–15 μm [6] that decreases with ongoing osseointegration [7, 8]. However, for the passive fit of implant-supported prosthetic restorations, accurate three-dimensional transfer of the intraoral implant position to the model cast is indispensable [9, 10]. Today, the inaccuracy of implant impression taking is compensated by intraorally bonded tertiary structures to achieve a tension-free, passive fit of the implant-supported prosthodontic restoration [11]. Therefore, improvements in the accuracy of implant impression taking are urgently required.

Different approaches have been investigated for improving the accuracy of implant impressions. Initially, implant impression taking involved individually preparation of prefabricated impression posts like natural teeth and then taking an impression. However, this technique was abandoned after a short time due to a lack of accuracy [9, 12–15]. Currently, the pick-up impression technique with conventional custom implant impression trays is recommended, especially when considering multiple implants [16].

To avoid the elaborate manual manufacturing process of conventional custom implant impression trays, customized impression trays with foils were developed. The foil tray was placed over the impression posts, and the foil was perforated at the respective regions of the impression posts to retrieve the impression post screws [17]. This is similar to the established conventional custom implant impressions of an open (pick-up) implant impression procedure, as required [18, 19].

In addition to the impression technique, improvements have been made to enhance the accuracy of the impression material. Depending on the number of implants and the angulation, polyethers and vinyl polysiloxanes are most commonly used in clinical practice [1, 4, 18, 20].

However, in recent years, the focus has been on improving digital implant impression taking with intraoral scanners to achieve the transfer accuracy of conventional custom implant impressions [21–23]. However, intraoral scanners still have limitations regarding the matching/stitching process resulting in reduced transfer accuracy for full-arch impression taking [24–28]. Moreover, conventional impression-taking methods are still widely used.

Nevertheless, intraoral scanners have not been the only innovation in dentistry in the last few years. Recently, digital devices such as additive manufacturing, commonly known in dentistry as three-dimensional (3-D) printing, have provided new options for manufacturing patient-specific individual impression trays [29–32]. The 3-D form freedom constitutes a huge advantage and should be emphasized. Especially the ability to adjust parameters such as the thickness of the layers and the influence of the openings in the impression tray could potentially affect accuracy, as described in the literature [33, 34].

Unfortunately, few studies have evaluated the accuracy of implant impressions using the 3-D printed customized impression trays to date [35–39]. However, to the best of the authors’ knowledge, there are no available data comparing the aforementioned implant impression techniques with the established study setup.

Furthermore, studies on the transfer accuracy are typically performed using best-fit superimposition methods. This often leads to incorrect results because best-fit algorithms determine the minimum distances between points that are not evaluated within a defined coordinate system. As shown in a previous investigation, an exact statement regarding the deviations is only possible through a three-dimensional evaluation within a coordinate system using a reference structure [27, 28, 40].

Therefore, the aim of the present study was to investigate whether chairside 3-D printed impression trays could increase the accuracy of implant impression taking using an established coordinate-based investigation method.

The following null hypothesis was formulated: there is no significant difference between the different impression techniques regarding the accuracy of implant impressions. The primary outcome was defined as the accuracy between the four impression trays. The secondary outcome was the implant position.

## Methods

On a master model of a partially edentulous maxilla with four implants in posterior region, different impression techniques were investigated: conventional custom impression tray (CIT), customized foil tray (CFT), chairside 3-D printed impression tray using the SHERA system (3DS), and the Primeprint system (3DP). Each impression technique was investigated separately, resulting in four study groups.

All experiments were conducted under laboratory conditions (room temperature, 23° C ± 1° C; humidity, 50% ± 10%).

**Fig. 1:**
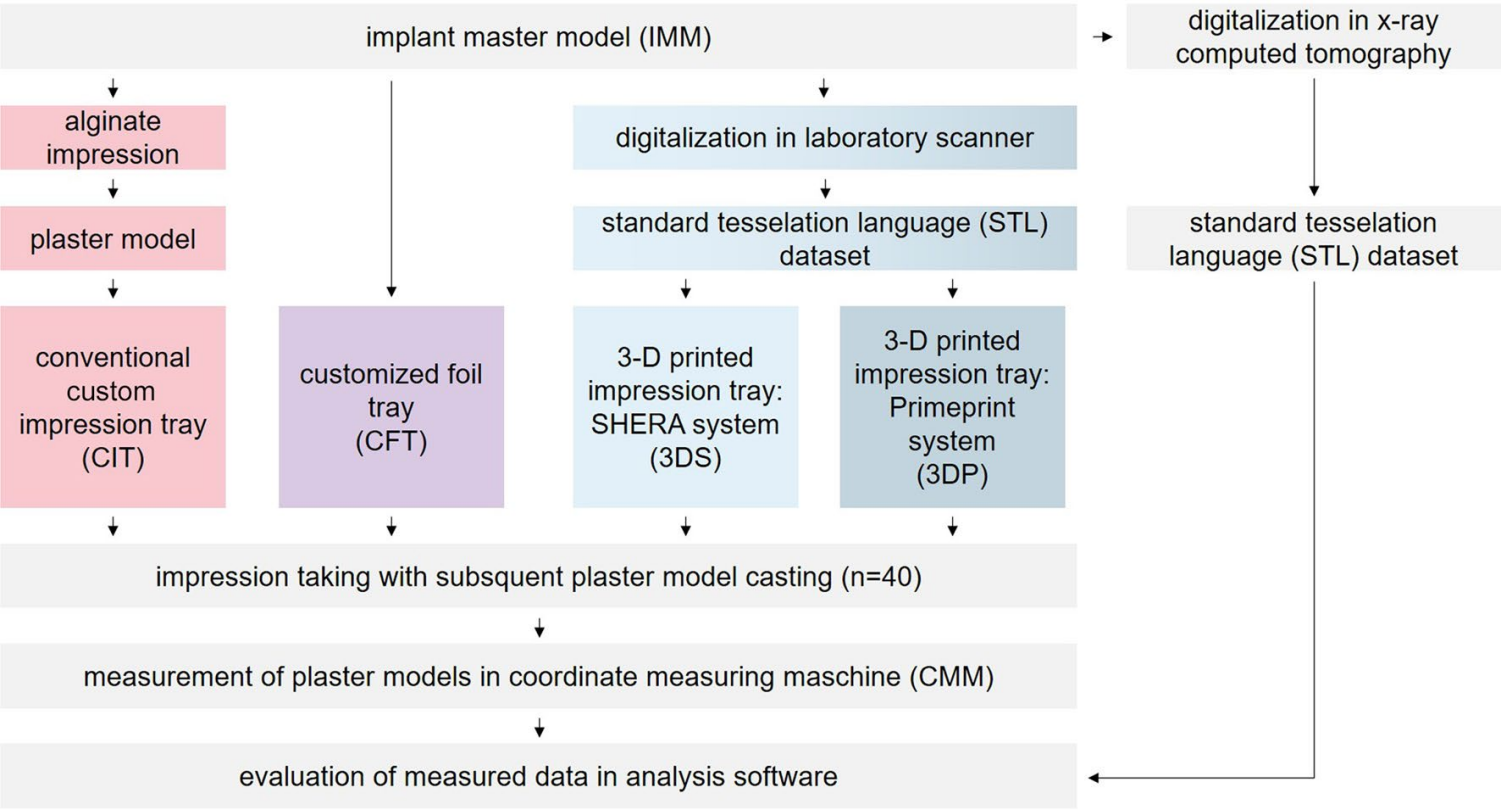
Flow scheme of the study protocol

For a better overview, Fig. 1 displays a flow scheme of the investigation.

### Implant master model and acquisition of reference dataset

**Fig. 2:**
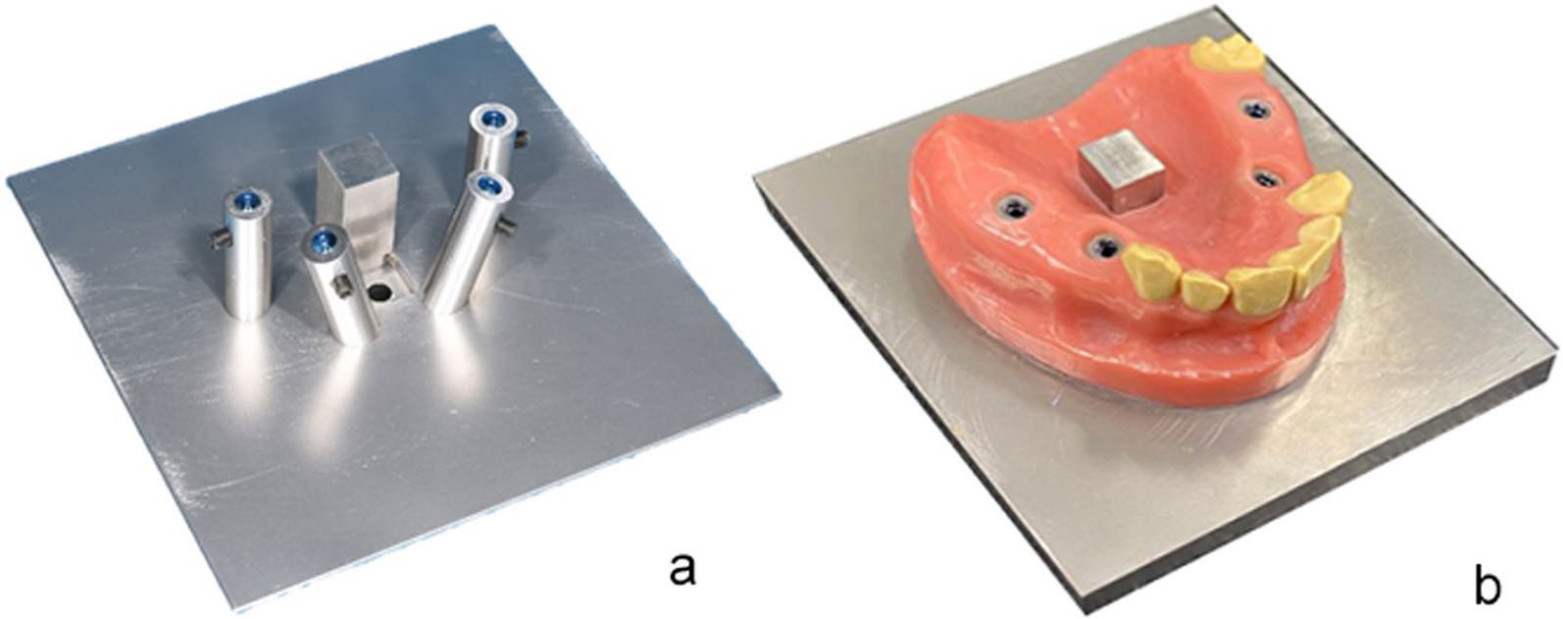
**a** IMM with implants #14/#24 and #16/#26. **b** Finalized IMM with model teeth #17,#13 to #23

An implant master model (IMM) of the maxilla, known from a previous study, was used as the patient equivalent [41]. On a stainless steel base plate (alloy 1.4301 [Ni–Cr], 100 × 100 × 15 mm), four stainless steel tubes were placed in the implant position of the first premolar (according to Federation Dentaire Internationale [FDI] schemes #14 and #24) and the first molar (#16 and #26, Fig. 2a). In each tube, an implant (4.1 mm diameter, 11.5 mm length, T3 non-platform switched tapered implants; Biomet 3i, Palm Beach Gardens, FL, USA) was adhesively luted (AGC-Cem Automix System, C. HAFNER, Wimsheim, Germany). The implants at positions #14 and #24 were inclined 15° in the buccal direction, while the implants were kept straight with 0° inclination at #16 and #26. In addition, a rectangular cube (10 × 10 × 20 mm) was placed at the center of the IMM perpendicular to the base plate to serve as a reference point. The IMM was finalized by modeling a partially edentulous maxilla with teeth in regions #17, #13, and #23 using pink and tooth-colored denture plastics (PalaXpress, Kulzer, Hanau, Germany; Fig. 2b).

**Fig. 3:**
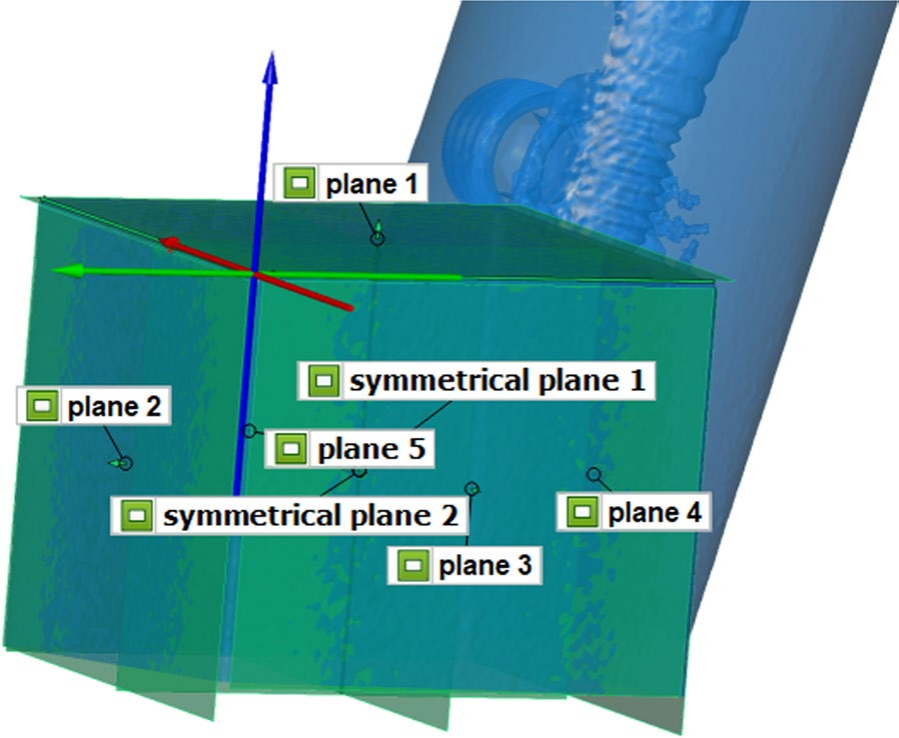
Reference cube of IMM with coordinate system: *x*-axis (red), *y*-axis (green), and *z*-axis (blue)

The IMM was digitized with an X-ray computed tomography (TomoScope S, Werth Messtechnik, Giessen, Germany; measurement parameters: 225 kV, 100 ms, 1 mm tin filter, 60 µm voxel size, 2200 sections, surface resolution < 6 µm, linear accuracy < 4 µm). On the resulting standard tessellation language (STL) dataset, the implant–abutment interface points (IAIPs) were determined for each of the four implants using WinWerth software (Werth Messtechnik). Finally, a coordinate system was created on the reference cube. The *z*-axis was defined as the intersection of the two symmetry planes of the outer surfaces of the cube. The line of intersection between the left outer plane and upper plane of the cuboid formed the *x*-axis. The intersection line between the rear, outer, and upper planes of the cuboid formed the *y*-axis. The origin was placed at the intersection of the *x*- and *y*-axes. All the coordinate systems for the measurements were created in the similar manner (Fig. 3).

Healing caps (ISHA42, Biomet 3i) were screwed into the four implants to manufacture impression trays.

### Conventional custom impression tray (CIT)

**Fig. 4:**
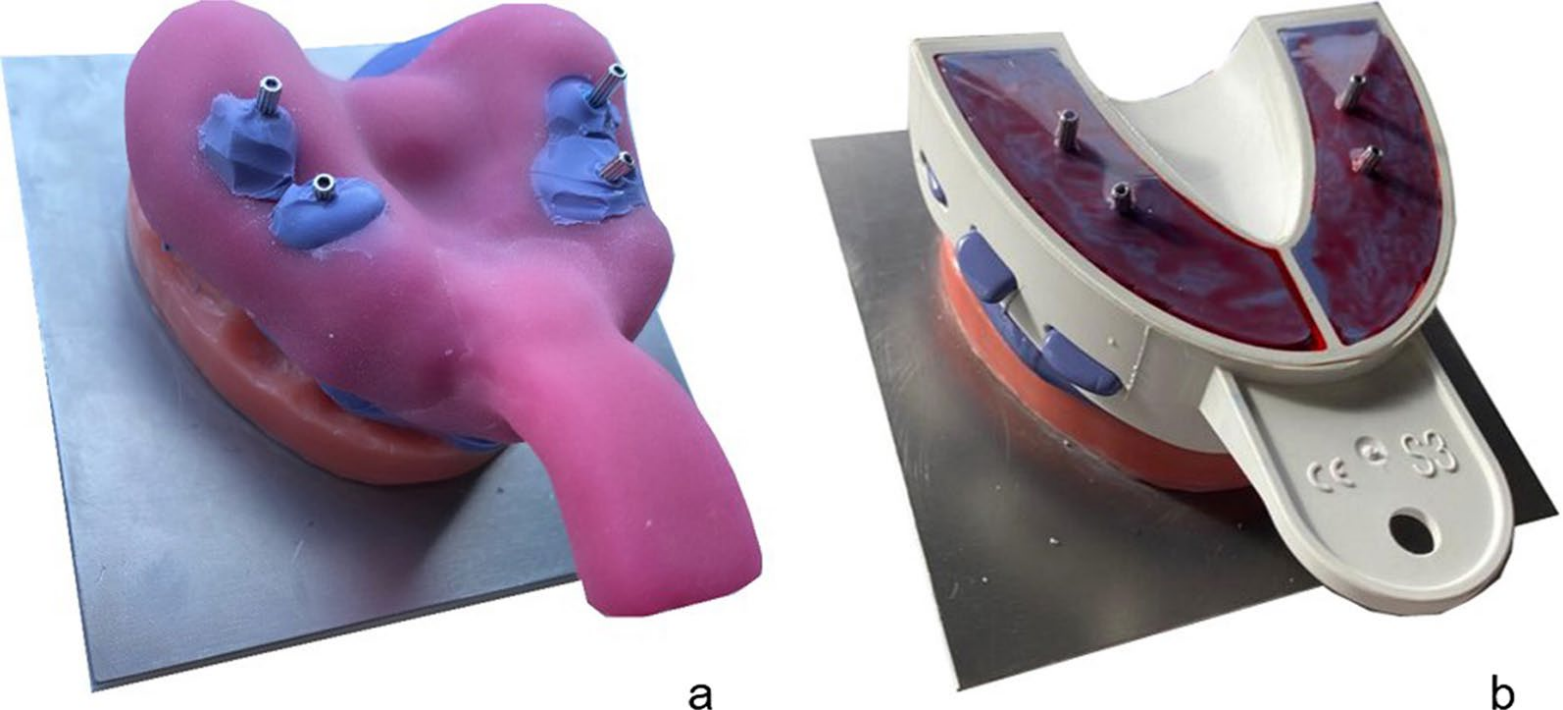
**a** Example of CIT and **b** CFT with impression posts in regions #14/#24 and #16/#26

To fabricate two CITs for open impression taking using the pick-up technique, an initial impression of the IMM with a stock metal tray and alginate (Cavex cream normal set, Cavex, Norden, Germany) was used. After manufacturing a plaster model (super-hard plaster type IV; Fujirock EP, GC, Leuven, Belgium), two CITs were fabricated using cold-cured polymethylmethacrylate (PMMA; C-plast, Candulor Dental, Rielasingen-Worblingen, Germany) with a layer thickness of 2.5 mm. In the implant position regions #14/#24 and #16/#26, a chimney-like opening with a diameter of 2.5 cm was designed, and a tray handle was placed in the anterior region (Fig. 4a).

### Customized foil tray (CFT)

In contrast to CTI, no elaborate manufacturing process is required for CFT (Miratray, Hager und Werken, Duisburg, Germany). The correct size of the CFT was determined (size 3 for the maxilla), and before impression taking, the foil was perforated with a dental probe in the region of the four impression posts (#14/#24 and #16/#26, Fig. 4b). According to the manufacturer's recommendations, the foil tray is designed for single use. Therefore, a new CFT was used for each impression.

### Chairside 3-D printed impression trays

Two different chairside workflows for manufacturing of 3-D printed impression trays were analyzed: the SHERA system (3DS, SHERA Werkstoff-Technologie, Lemförde, Germany) and the Primeprint system (3DP, Dentsply Sirona, Bensheim, Germany). To simulate a close clinical study setup, the IMM was digitized using a laboratory scanner (D2000, 3Shape, Copenhagen, Denmark).

#### SHERA system (3DS)

**Fig. 5:**
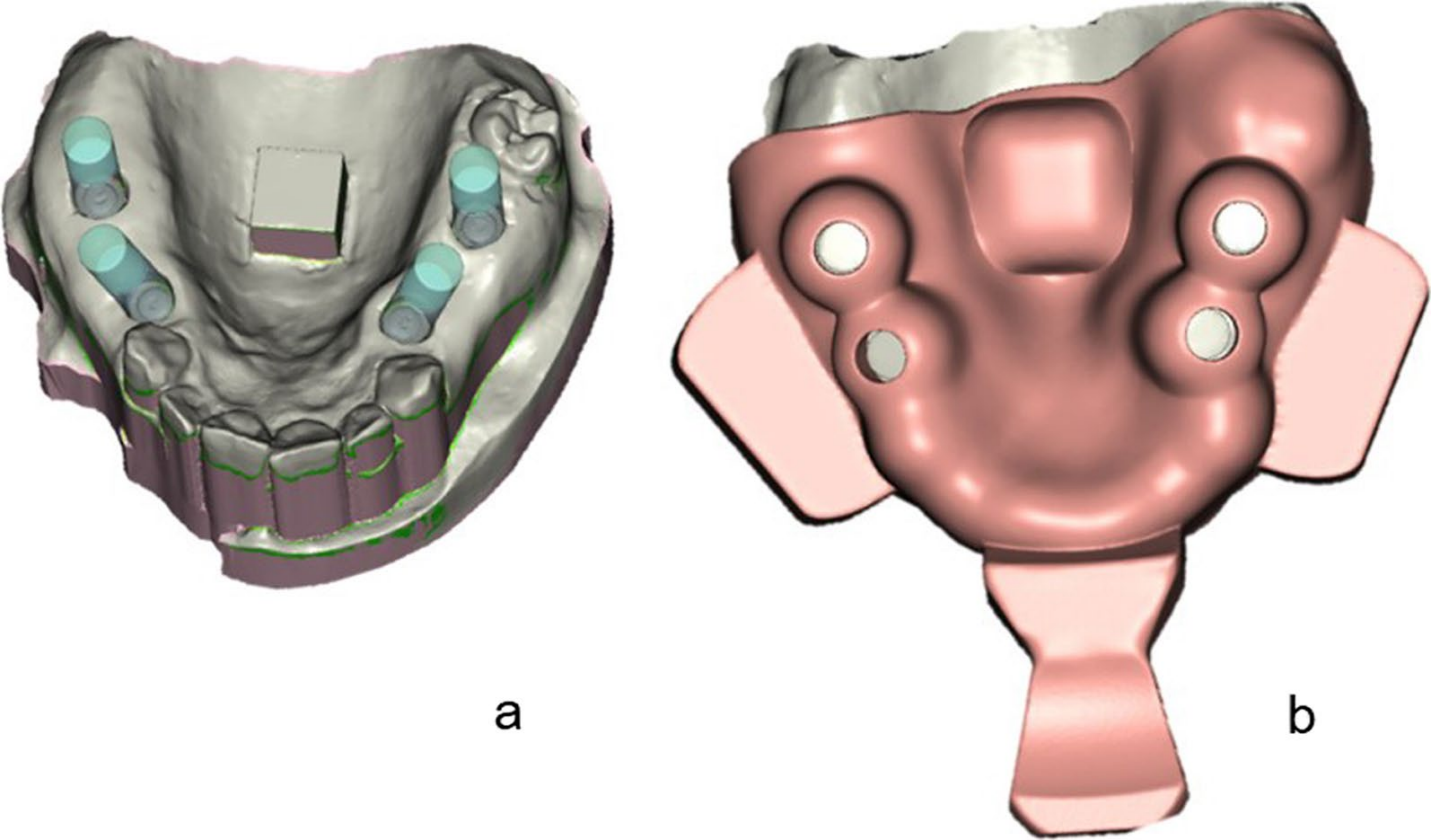
**a** Example of the 3DS with automatically calculated chimney-like tray openings and **b** the designed tray

First, the STL dataset of the IMM was imported into the computer-aided design (CAD) software SHERAeasy-base (version 2.0; SHERA Werkstoff-Technologie, Lemförde, Germany). The following features were selected: impression type, implant impression; impression material, polyether; implant system, Biomet 3i; and height of the healing cap, 2 mm. Further data on the respective implant systems were stored in the digital library of the software. Thus, the implant axis and the corresponding position of the chimney-like tray openings were calculated automatically, as well as the direction of insertion for the tray and the block out of the undercuts (Fig. 5a; chimney diameter 10 mm, thickness of the tray 3 mm).

Next, the contours of the trays were determined. Finally, buccal bars were designed in addition to a tray handle to facilitate the removal of trays from the IMM, and the CAD dataset was exported (Fig. 5b).

The computer-aided manufacturing (CAM) software Netfabb (version 2022, Autodesk, Munich, Germany) was used for nesting the CAD dataset. As two trays had to be fabricated, the CAD dataset was duplicated. Therefore, two trays were placed on the virtual printing platform in the software and support structures were added. The final dataset was exported to rapid-shape format, and transferred to the digital light-processing SHERAPrint 30 3D printer (SHERA Werkstoff-Technologie). For additive manufacturing, the light-curing pink-colored resin SHERAprint-tray clear (SHERA Werkstoff-Technologie) was used. After completing the printing process, the trays were manually detached from the platform. Followed by a post-processing cleaning in the SHERAprint-wash cleaning and drying unit (SHERA Werkstoff-Technologie), the support structures were cut off and trays were post-polymerized in the SHERA print-cure light-curing unit (SHERA Werkstoff-Technologie).

#### Primeprint system (3DP)

**Fig. 6:**
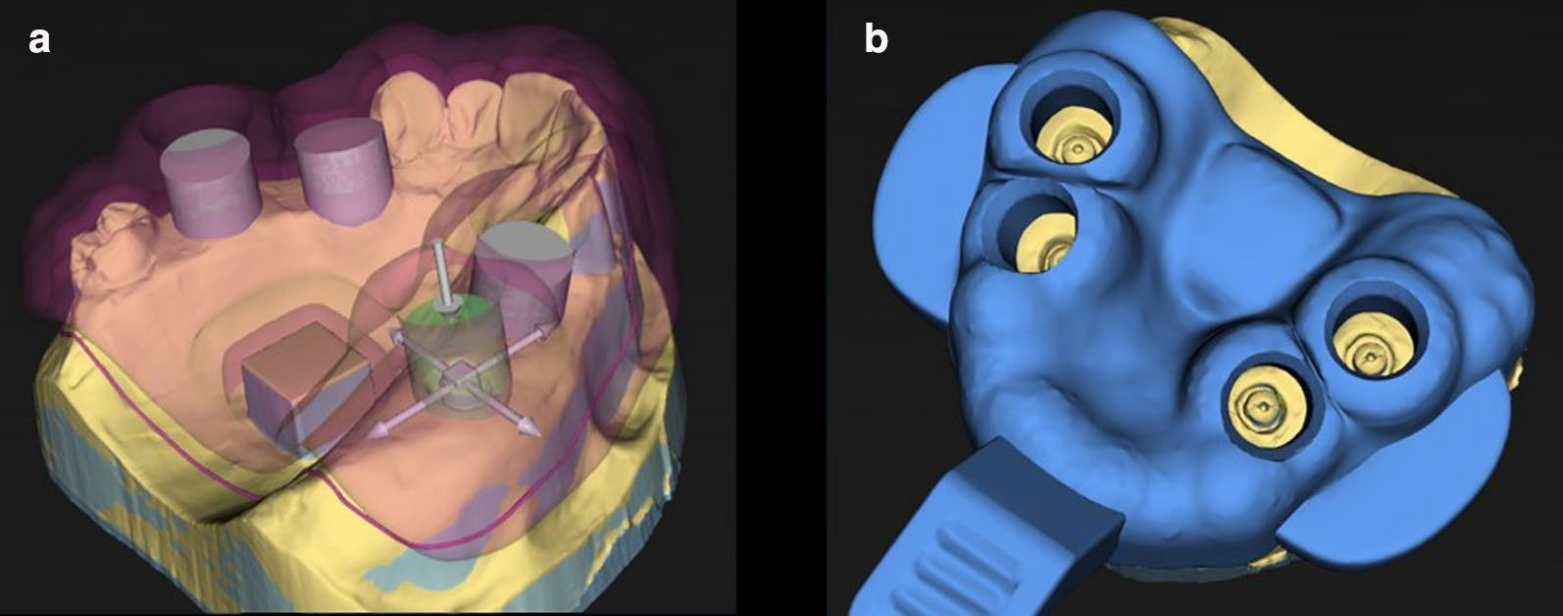
**a** Example of the 3DP with manually designed chimney-like tray openings and** b** the designed tray

For the manufacturing of the chairside 3-D printed impression trays with the Primeprint system, the STL dataset of the IMM was imported into the inLab CAD software (version SW 22.1.1, Dentsply Sirona). After positioning the dataset in the coordinate system of the inLab CAD software, a virtual model of the IMM was created. The inLab splint software (version 22.0.3, Dentsply Sirona) was opened using inLab CAD software. In contrast to the SHERA system, all steps of the tray design had to be selected manually, except for the automatically determined direction of insertion of the tray and the block out of the undercuts. Therefore, a tray contour, gingiva former height of 2 mm, implant axis, and chimney-like openings were designed for the impression posts (Fig. 6a). The diameters of the openings were selected based on the implant and impression post used (10 mm in the present study design). The thickness of the impression tray was 3 mm. In the final design process, buccal bars were added next to the tray handle to facilitate the removal of the tray from the IMM (Fig. 6b).

For nesting the CAD dataset, the inLab CAM software (version 22.2.0, Dentsply Sirona) was used. As two trays had to be fabricated, the CAD dataset was duplicated. Two trays were placed on the virtual printing platform in the software and support structures were added. According to the manufacturer, the printing process was performed using the material Primeprint Tray (Dentsply Sirona). Finally, the tray was automatically cleaned and post-polymerized in the Primeprint Post Processing Unit (PPU, Dentsply Sirona). After the tray was detached from the platform, the support structures were removed manually.

**Fig. 7:**
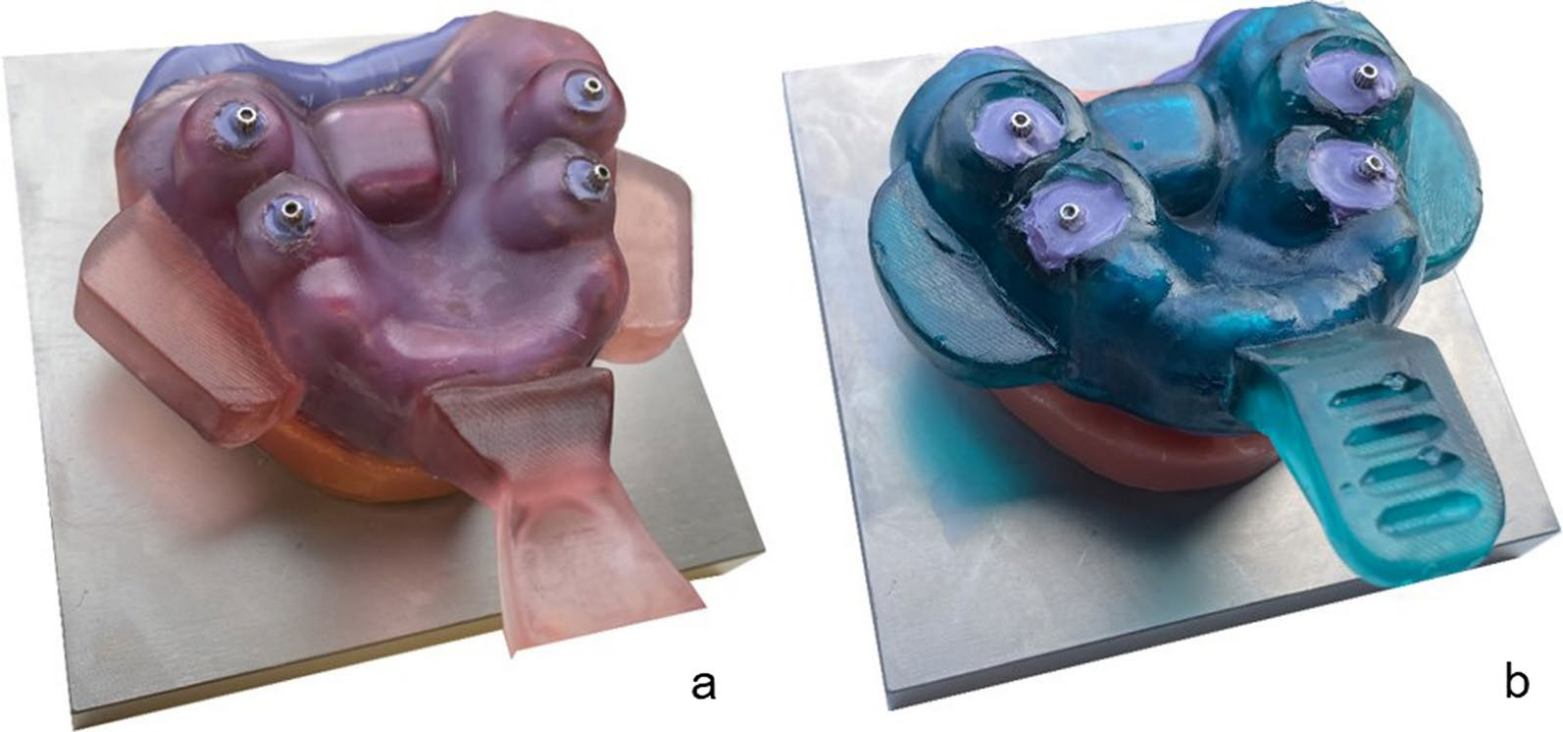
**a** Example of 3DS and **b** 3DP with inserted impression posts in regions #14/#24 and #16/#26

Figure 7 shows an example of the 3-D printed impression trays.

### Implant impression taking

Ten implant impressions were obtained from each of the four study groups. After five impressions, the trays in the CIT, 3DS, and 3DP groups were replaced, whereas in the CFT group, a new customized tray was used for each impression.

All the trays were coated with a thin layer of Polyether Adhesive (3 M, Neuss, Germany). For implants in positions #16 and #26, implant system-specific impression posts with anti-rotation protection ((IIIC42—non-hexed, Biomet 3i) were used, whereas for implants in regions #14 and #24, impression posts without anti-rotation protection (IIIC41, Biomet 3i) were applied. All impression posts were tightened with a torque of 10 Ncm according to the manufacturers’ instructions.

Polyether (Impregum Penta, 3 M) was used as the impression material and automatically mixed with the corresponding Pentamix 3 mixing device (3 M). After a setting time of 6 min, the screws of the impression posts were unscrewed, and the impression tray with the impression posts embedded in the impression material were removed from the IMM.

### Fabrication of the plaster models

To ensure recovery of the polyether impression material, all impressions were stored for at least 45 min, and laboratory analogs (H51, H-series, nt-Trading, Karlsruhe, Germany) with a diameter of 4.1 mm were screwed with a torque of 10 Ncm into the impression posts in the implant impression to reproduce the implant position during model fabrication.

Type IV plaster (Fujirock EP, GC, Leuven, Belgium) was used to fabricate the plaster models according to the manufacturer’s instructions. The plaster models were demolded from the impressions after 60 min. Model trimming was omitted because of the potential dimensional changes in the models due to water absorption. Prior to subsequent measurements, the models were stored for 7 days.

### Measurement and evaluation of the plaster models

The 40 plaster models were measured using the coordinate measuring machine (CMM) CNC Thome RAPID (Thome Präzision, Messel, Germany) with the corresponding measuring software Metrolog X4 (version 10, Metrologic, Meylan, France). To determine the implant position on the plaster casts, scanbodies (H-series, nt-Trading) with a polyetherketone (PEEK) base and titanium wing surfaces were screwed into laboratory analogs with a torque of 10 Ncm. The scanbodies used were measured individually in the CMM prior to the study to obtain the exact length and determine the implant position as accurately as possible during evaluation.

The first measurement was performed manually and recorded using the measurement software as a measurement and inspection template, based on which the measurement process was repeated five times. First, all five planes of the reference cube (Fig. 1) were probed at four points using a 3-mm-diameter ruby head probe (Renishaw, Pliezhausen, Germany). Subsequently, these planes were circularly measured automatically at 7000 points. Next, the scanbodies were probed with a 1.5-mm-diameter ruby head. The upper surface of each scanbody was probed as a plane with three points and measured in an automatic circle to avoid faulty touches. Subsequently, a cylinder was constructed by probing the scanbodies at 12 points.

After all the elements were measured, a coordinate system with an origin point was created on the reference cube. The points between the implant and abutments (implant-abutment interface points/IASPs) were constructed by shifting the intersection points of the cylinder and planes of the scanbodies using the previously determined length of the scanbodies. After each pass, the collected data were saved and arithmetically averaged for each model. The determined *x*-, *y*-, and *z*-coordinates of the IASP were imported into the inspection software GOM Inspect 2022 (GOM, Braunschweig, Germany) and aligned to the original coordinate system of the IMM. Subsequently, the distances in the *x*-, *y*-, and *z*-directions between the respective determined and reference points were constructed and measured to obtain the deviation from the master model.

### Statistical analysis

Statistical analyses were performed using SPSS 26 (IBM, Armonk, NY, USA) with an alpha error of 5%. To investigate whether the impression technique and implant position differed significantly in terms of absolute deviation, a two-factor 4 × 4 ANOVA (analysis of variance (ANOVA) was performed. In addition, a robustness analysis was performed to exclude the possibility of distortions in the statistical analysis owing to outliers. Only a marginal difference was observed between the results of all cases and those without outliers. Because clear variance heterogeneity was observed, the model was calculated using MIXED (estimation method REML, degrees of freedom according to Satterthwaite).

The multiple pairwise comparisons were corrected for alpha error accumulation according to SIDAK.

Data are presented as boxplot diagrams. Trueness (mean) and precision (SD) are reported according to the International Organization for Standardization (ISO) 5725 [42].

## Results

**Fig. 8:**
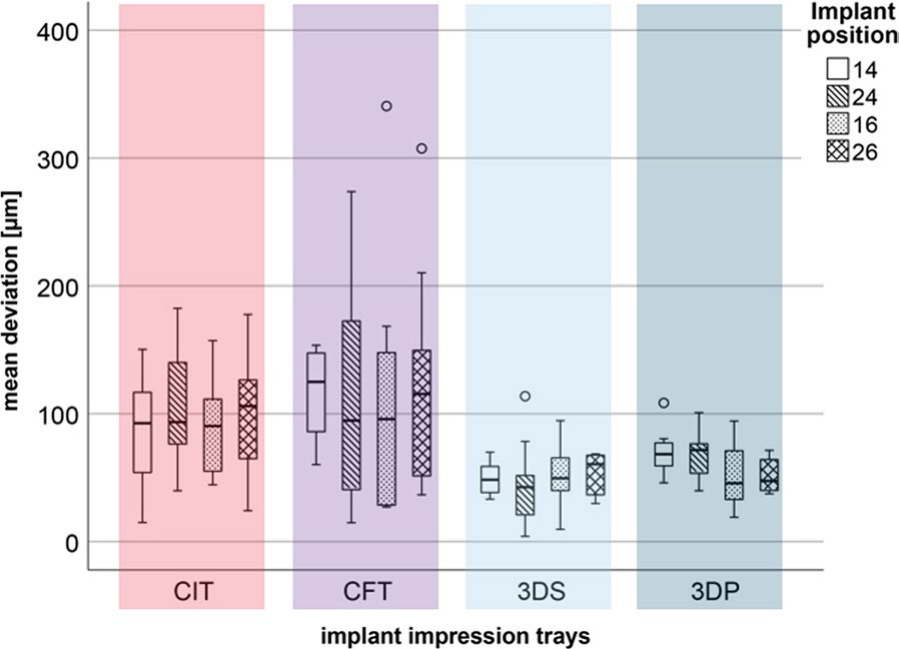
Boxplot diagram of the mean deviation for CIT, CFT, 3DS, and 3DP

The results of the mean deviations between the reference dataset of digitalized IMM in x-ray computed tomography and the measurements of the four study groups distributed to the implant positions are displayed in Fig. 8.

Table 1 presents the *p*-values for trueness and precision between the study groups.

**Table 1:**
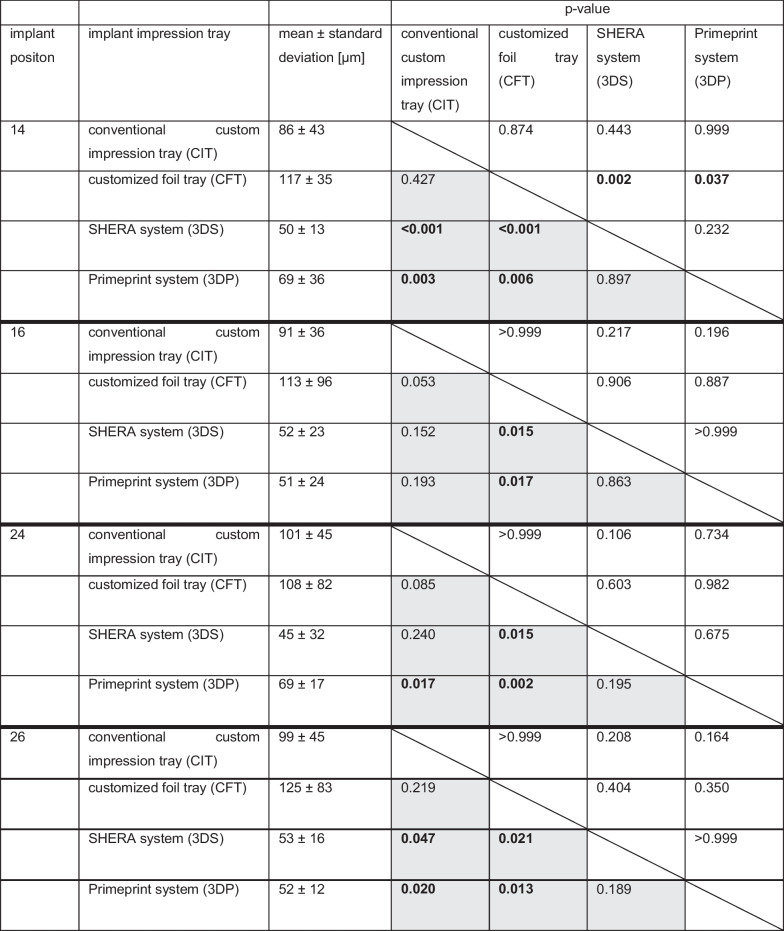
*P*-values between methods for trueness and precision

Trueness upper right side/precision lower left side; significant differences are indicated in bold type

Within the model setup, the deviations were independent of the implant position. The variations in each study group were constant within the study groups. Overall, the chairside 3-D-printed impression trays (3DS and 3DP) exhibited the best results, followed by conventional custom impression trays (CIT). Implant impression taking using a customized foil tray (CFT) exhibited the lowest accuracy. Significant differences were observed regarding the trueness for implant position #14 between the 3DS/3DP and CFT groups (*p* < 0.05). In terms of precision, significant differences were found for all implant positions between 3DS/3DP and CFT (*p* < 0.05). However, this was only partially detected between the CIT and 3DS/3DP groups.

Therefore, the null hypothesis was partially rejected, as differences between the different impression techniques were found in terms of transfer accuracy.

## Discussion

Even though attention on implant impressions is currently focused on improvements in digital implant impression taking with intraoral scanners, developments regarding conventional methods should not be neglected. In addition to the limitations of intraoral scanners, many dentists still use conventional methods to obtain implant impressions. Furthermore, a combination of new digital and established conventional methods may improve treatment methods, as shown by the results of this study.

A comparison of the results of the present study with those of other studies needs consideration of the reference cuboid and measurement strategy. The exact three-dimensional deviations can be calculated by superimposition within the coordinate system. This must be taken into account when comparing with the results of other studies, as shown previously [40]. The results are directly dependent on the measurement strategy used. In contrast to the present study, higher accuracies were achieved in a study by Izadi et al. [43] regarding the impressions of conventional custom individual implant trays. However, in this study, no separate reference body was used, as only one of the three implants examined served as a reference. Furthermore, the distance between the implants was significantly smaller because all implants were located exclusively in the anterior region. Moreover, the implants were placed parallel to each other and were not angulated, as in the present study. In addition, there was no residual dentition, which is particularly important considering the lack of undercuts and the typically associated lower deformations caused by removing impressions.

Tohme [44] and Ribeiro [45] demonstrated higher overall deviations. However, this might be related to the measurement method used. In contrast to the present study, the measurements were performed with the aid of an optical scanning system and not by a CMM.

In contrast, the results obtained by Rech-Ortega et al. [46] were similar to those of the present study. The deviations ranged from 20 to 123 µm. In addition, this was corroborated by D'Haese, who showed average deviations of 86 µm [47].

To the best of our knowledge, the foil impression trays investigated have only been used in one previous study [17]. The accuracy achieved was just below the results of the present study. Notably, the precision of approximately 65 µm was particularly high. This may be explained by the existing flexibility and associated deformation when the impression tray was removed from the model. Furthermore, a different method was used to measure the gap between implant positions by Marotti et al. [17], which makes it difficult to compare the results.

Compared to the other impression methods, impressions with the 3-D-printed trays showed the highest accuracy. However, the studies available for comparison are scarce. The results obtained by Liu et al. [36] were similar to those obtained by the present study. However, the individual impression posts were additionally splinted using a 3-D printed bar prior to impression taking. More precise results were obtained by Revilla-Leon [48], who used the implants at an inclination of 10° instead of 15° as used in the present study. Furthermore, impressions were superimposed using a best-fit algorithm and not evaluated using a coordinate-based measurement method.

Yang et al. [39] did not find any significant difference between the impression accuracy of additively manufactured and conventional impression trays. However, in contrast to the present study, gap measurements were performed between the implant and reference key using an optical microscope. Gap widths of 31 ± 3 μm were obtained for the additively manufactured tray and 32 ± 3 μm for the conventional tray. Owing to the different measurement methodologies and the design of the in vivo study, the direct comparability of the results is limited.

The main advantage of conventional custom impression trays is the uniform distance to the model, and thus to the gingiva and teeth, realized by CAD/CAM fabrication. Directly connected to this is a circular, uniform, chimney-like enclosure of impression posts, whereby a circular, uniform layer thickness of the impression material can be achieved. This can positively influence the possible shrinkage, as shrinkage can occur uniformly on all sides, and it can be assumed that lower stresses and restoring forces occur after tray removal [16].

In contrast to the Primeprint system, the SHERA system uses an automatically design process based on a digital implant library. This might position the chimney-like openings more precisely than the manually workflow of Primeprint system. This may explain why the results of the SHERA system were slightly better, although no statistically significant differences were observed.

In summary, this study demonstrates that further developments in digitization and 3-D printing can further improve conventional impression taking processes. This is emblematic of the fact that digitization is not about the forced conversion of all previous conventional manufacturing paths into digital paths, but about the corresponding sensible use of new technologies to be able to achieve better results overall.

**Fig. 9:**
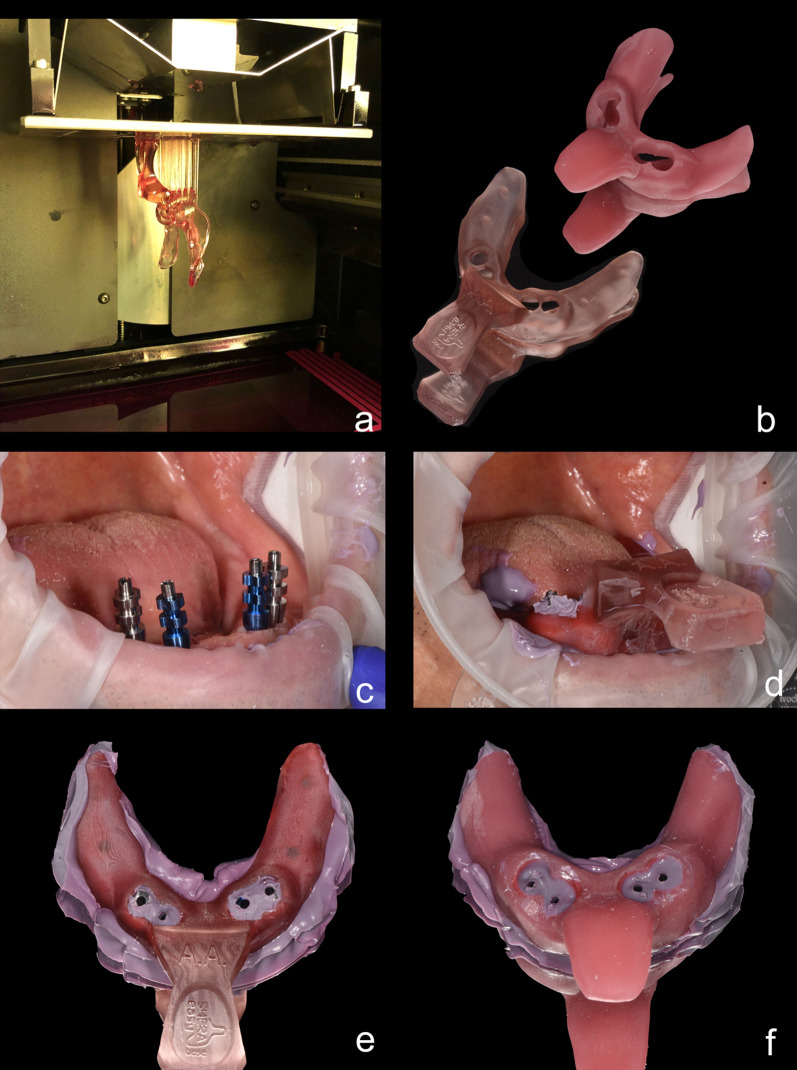
Proof of principle in one patient to better illustrate the daily practice. **a** A 3-D printed tray with SHERA system displaying before and **b** after post-processing compared to manually manufactured conventional individual impression tray. **c** Intraoral situation with screwed impression post and** d** impression taking with 3-D printed tray and polyether impression material. **e** Comparison between 3-D printed tray and **f** conventional custom impression tray after impression taking from top view

A clear limitation of this study is its in vitro design. However, the new technologies require highly standardized setups. Nevertheless, we decided to evaluate the presented technique on a patient to prove that the concept of 3-D printed trays is clinically applicable in daily practice (Fig. 9). Therefore, we performed two implant impressions with one conventional custom impression tray and one 3-D printed SHERA system tray. As reference structure for further evaluation, we used the methodology of Schmidt et al. [40]. Higher accuracy was found for 3-D printed SHERA system compared to conventional custom impression, as already shown in the present in vitro study.

It is also important to note that 3D printed trays are currently more time-consuming to manufacture compared to the custom foil tray and the conventional custom impression tray. However, in our experience, 3D printed trays require only half the amount of impression material compared to the other two trays.

Further research, particularly in the form of a systematic clinical study, should be conducted to make a more concrete statement about the clinical transfer accuracy of 3D printed trays.

## Conclusions

Based on the present results, it was shown for the first time that significantly higher transfer accuracies could be achieved by applying 3-D-printed impression trays for implant impression taking. This is particularly relevant for daily practice. From the present results, it can be concluded that if a 3-D printer is available in the practice or dental laboratory, it can be recommended for the manufacturing of patient-specific impression trays, to improve the transfer accuracy of conventional implant impression taking for a better fit of prosthodontic restoration. Evaluation with the aid of a defined reference cuboid within a coordinate system revealed differences that could be masked by a best-fit superimposition. Future studies with clinical applications should follow a single patient case to validate the results of a systematic clinical study.

## Data Availability

Data are provided by corresponding author on reasonable request.

## Abbreviations

3-D: Three-dimensional
3DS: Chairside 3-D printed impression tray with SHERA system
3DP: Chairside 3-D printed impression tray with Primeprint system
CAD: Computer-aided design
CAM: Computer-aided manufacturing
CFT: Customized foil tray
CIT: Conventional custom impression tray
CMM: Coordinate measuring machine
FDI: Federation Dentaire Internationale
IAIP: Implant Abutment Interface Point
IMM: Implant master model
ISO: International Organization for Standardization
PMMA: Polymethylmethacrylate
STL: Standard tessellation language
